# Anticardiolipin Antibody Determination to Guide Q Fever Treatment

**DOI:** 10.7759/cureus.51840

**Published:** 2024-01-08

**Authors:** Diogo Costa Oliveira, José Guilherme Assis, Fernanda Linhares, Paulo Carrola

**Affiliations:** 1 Intensive Care Unit, Centro Hospitalar de Trás-os-Montes e Alto Douro, Vila Real, PRT; 2 Internal Medicine, Centro Hospitalar de Trás-os-Montes e Alto Douro, Vila Real, PRT

**Keywords:** zoonosis, endocarditis, anticardiolipin antibodies, hydroxychloroquine, coxiella burnetii, q fever

## Abstract

Query (Q) fever is a worldwide infectious disease with acute and chronic manifestations caused by *Coxiella burnetii*. The clinical manifestations are so variable that the disease is often only diagnosed if systematically considered as a differential diagnosis. Here, we present a case of a 39-year-old man who lived in a countryside house, with cattle and sheep in his field, with acute Q fever hepatitis with the typical granulomatous arrangement in the liver biopsy. The diagnosis was confirmed by *polymerase chain reaction* (PCR) assay in a serum sample and the presence of phase II antibodies. Anticardiolipin antibody (aCL) determination at diagnosis of acute Q fever and during follow-up was made to persecute early identification and to guide the treatment and prophylaxis of possible complications, such as endocarditis.

## Introduction

Query (Q) fever is a worldwide infectious disease caused by *Coxiella burnetii*, a strictly intracellular Gram-negative bacteria that lives and multiplies in the phagolysosomes of infected cells [1]. Cattle, sheep, and goats are commonly infected, and humans often become exposed by breathing in dust contaminated with infected animal body fluids [2]. Persons at greater risk include farmers, abattoir workers, and veterinarians [3]. The incubation period of the disease is two to three weeks after exposure.

*C. burnetii* infection in humans is usually asymptomatic or manifests as a mild disease with spontaneous recovery. The clinical manifestations are so vast that the disease is often only diagnosed if systematically considered as a differential diagnosis [2]. Prompt diagnosis and appropriate treatment shortens the illness and reduces the risk for severe complications.

In this case report, we discuss the use of anticardiolipin antibody (aCL) as an early marker to identify progression to Q fever complications and review its use in clinical practice to guide the treatment.

## Case presentation

We present a clinical case of a 39-year-old man with no pre-existing medical conditions admitted for the study of a one-week history of fever (38.5ºC) and general malaise with myalgia, overall weakness, and chills. He denied present or recent respiratory, gastrointestinal, or urinary symptoms. On physical examination, no skin lesions and no palpable adenopathy were identified; cardiopulmonary auscultation was normal; hepatomegaly was noted with smooth, regular borders, 5 cm below the costal margin, with no splenomegaly.

Initial lab tests showed leucocytosis of 12,45x10^3^/µL with a left neutrophil shift and elevated C-reactive protein 6.95 mg/dL (N: <0.5 mg/dL); hemoglobin and platelet count were normal; aspartate aminotransferase was 195 U/L (N: <40 U/L), alanine aminotransferase was 205 U/L (N: <41 U/L), gamma-glutamyl transferase was 502 U/L (N: 10-49 U/L), and alkaline phosphatase was 124 U/L (N: 40-130 U/L) with an increased total bilirubin of 2.1 mg/dL (N: <1.2 mg/dL) due to the conjugated form. Chest film showed no alterations. Urine and blood cultures were negative (see Table 1).

**Table 1:** Initial laboratory results AST: aspartate transaminase, ALT: alanine transaminase, GGT: γ-glutamyl transferase, FA: fatty acid

	Result	Reference values
Hemoglobin	14.70 g/dL	13.0–18.0
Leucocytosis	12,45 x 10^3^/µL	4.0–11.0
Platelet count	257 x 10^3^ /uL	150–400
Serum creatinine	0.70 mg/dL	0.7–1.3
Ureia	25 mg/dL	<50
Sodium	129 mEq/L	135–147
AST	195 U/L	<40
ALT	205 U/L	<41
GGT	502 U/L	10-49
FA	124 U/L	40-130
Total bilirubin	2.10 mg/dL	<0.1
Direct bilirubin	1.90 mg/dL	<0.3
Lipase	42 U/L	13-60
C-reactive protein	6.95 mg/dL	<0.5
Serum PCR C. burnetti	Positive	
Phase I IgM Antibodies *C. burnetti*	1600	Negative: <50
Phase I IgG Antibodies* C. burnetti*	<200	Negative: <200
Phase II IgM Antibodies* C. burnetti*	<50	Negative: <50
Phase II IgG Antibodies* C. burnetti*	<200	Negative: 200

In our study of a fever without an initially obvious etiology, the patient’s epidemiological background guided subsequent investigation found: he lived in a countryside house, with cattle and sheep in his field, and frequently assisted neighbors with calving. A zoonosis panel was requested with a positive DNA PCR assay for *Coxiella burnetii*. Phase I IgM antibodies for *C. burnetti* were positive at 1600 (positive if ≥50); phase I IgG and phase II antibodies were negative.

**Figure 1:**
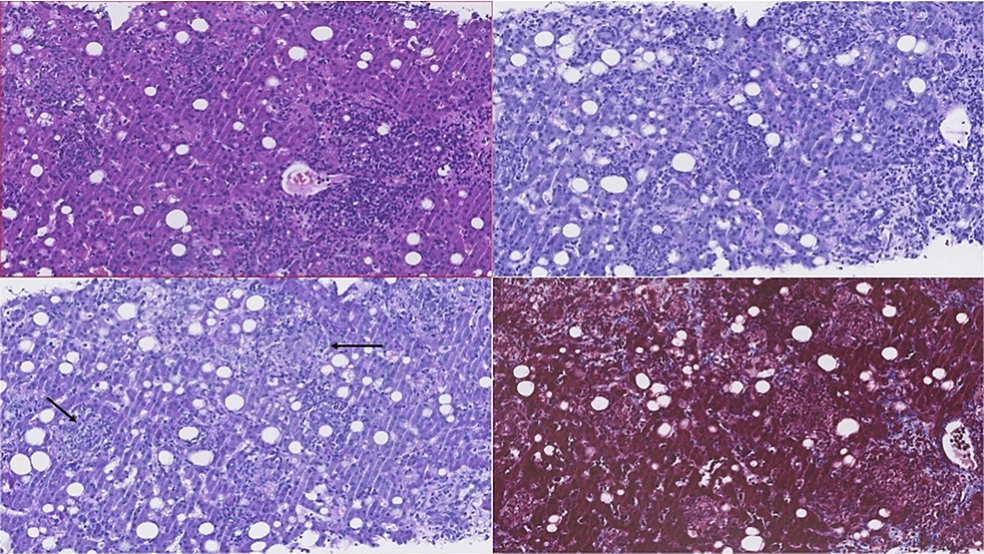
Histological examination of liver biopsy Histological examination of slides prepared with H&E staining and periodic-acid-Schiff (PAS) staining. Masson's trichrome shows liver tissue with a globally preserved lobular architecture and moderate to intense inflammatory infiltrate, with lymphocytes, plasmocytes, numerous polymorphonuclear neutrophils, and frequent epithelioid histiocytic cells with a vaguely granulomatous arrangement (thin black arrow), without necrosis.

**Figure 2:**
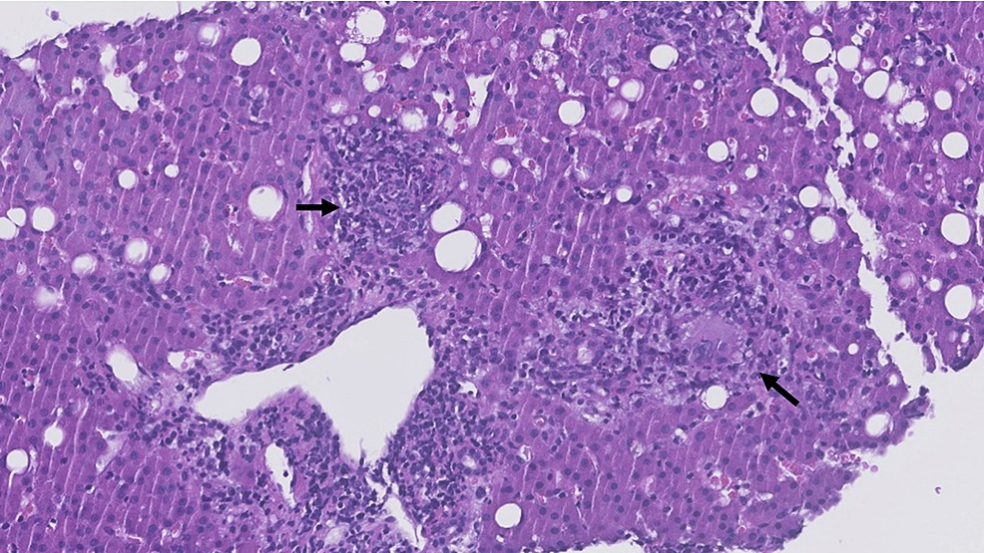
Granulomatous arrangement in H&E staining of liver biopsy H&E staining amplification (x40) showing the vaguely granulomatous arrangement (thick black arrow). Although typical fibrin-ring lipogranulomas were not identified, the findings are compatible with Q fever hepatitis.

At this stage, we started the patient on doxycycline 100 mg b.i.d. Abdominal ultrasound confirmed hepatomegaly of 19 cm. Liver biopsy revealed moderate to intense polymorphic, portal and lobular inflammatory infiltrates, and frequent epithelioid histiocytic cells with a granulomatous arrangement, without necrosis (see Figure 1 and Figure 2).

Two weeks later, the aCL became available: IgG aCL was normal at 9.8 GLP-U/ml (N: negative <10 GPL-U/ml, undetermined 10-40 GPL-U/ml, positive >40 GPL-U/ml); IgM aCL was elevated at 379.0 MPL (N: negative <7 MPL, undetermined 7-10 MPL, positive >10 MPL). Since the patient sustained fever and malaise, with elevated anticardiolipin antibodies, we decided to start hydroxychloroquine 200 mg t.i.d. and continued our investigation to exclude possible complications, especially endocarditis. A transthoracic echocardiogram (TTE) revealed no valvopathy or vegetation, with further confirmation on transesophageal echocardiography (TEE) performed one week later. 18F-FDG-PET/CT scan revealed hypermetabolic densifications in subpleural areas in the lower lobe of both lungs and mild bilateral pleural effusion but no valvular hypermetabolic foci.

**Table 2:** Evolution of anticardiolipin antibodies in the course of the disease The weeks refer to the date when the blood sample was drawn after the hospital admission date. Reference values: negative <10, undetermined 10-40, positive >40.

	Week 1	Week 4	Week 6	Week 12
IgG anticardiolipin antibody	9.8 GPL-U/ml	8.4 GPL-U/ml	4.0 GPL-U/ml	1.4 GPL-U/ml
IgM anticardiolipin antibody	379 MPL-U/mL	122 MPL-U/mL	59 MPL-U/mL	2.4 MPL-U/mL

The clinical evolution was favorable, with sustained apyrexia after initiating hydroxychloroquine. He was seen in the outpatient clinic two, four, and eight weeks after discharge, with decreasing IgM aCL, as shown in Table 2, and normalization of liver enzymes at the four-week follow-up consultation. Doxycycline and hydroxychloroquine were suspended upon normalization of the aCL, completing six weeks of treatment.

## Discussion

The French National Referral Centre defines acute Q fever as clinical symptoms (fever and/or hepatitis and/or pneumonia) with serologic criteria for phase II IgG levels ≥200 and phase II IgM levels ≥50, seroconversion, or a positive PCR assay [4]. PCR is of high specificity, with higher sensitivity when applied before seroconversion. Phase II antibodies appear between weeks 2 and 3 after the onset of symptoms [5].

In the hepatitis form of presentation of Q fever, liver biopsy presents with characteristic "doughnut-like" granulomas [6]. Although we could not isolate these in our case, we identified infiltrates with the granulomatous arrangement, in agreement with the diagnosis of Q fever.

Endocarditis is another feared complication of Q fever, which concerns fewer than 5% of patients [7]. The prolonged fever associated with the *C. brunetti* infection may act as a confounding factor and delay the beginning of investigations of possible complications. Recent literature has suggested aCL determination at diagnosis as an early, discriminating, and predictive biomarker of progression to Q fever endocarditis [4]. Some authors suggest TEE if the patient is male, over 40 years of age, aCL >60 GPLU, and a negative or inconclusive TTE [8]. Other authors suggest that in all patients >40 years of age and patients without significant valvulopathy on initial transthoracic echocardiography but with raised IgG aCL levels (>75 GPLU), a transoesophageal echocardiogram (TOE) should be performed to rule out undiagnosed valvulopathy [4]. 

Positive aCL has been reported in six rare complications of acute Q fever: hemophagocytic syndrome, alithiasis cholecystitis, meningitis, thrombosis, acute Q fever endocarditis, and hepatitis [9]. Recent literature suggests that aCL should be systematically assessed in acute Q fever, and hydroxychloroquine should be added to doxycycline in selected patients with increased aCL to prevent progression to endocarditis [10]. We decided to do this ourselves. Although initial TTE showed no endocarditis, our patient maintained a persistent fever. Since aCL was elevated, an 18F-FDG-PET/CT scan was done to identify possible complications, but it did not reveal any new relevant findings. The patient was then seen in the outpatient clinic with a favorable evolution and normalization of the aCL six weeks later, completing six weeks of treatment with doxycycline and hydroxychloroquine, with no readmission in the following year.

Cléa Melenoitte and colleagues seven separated the treatment of acute Q fever into three clinical entities: acute Q fever without complicated focus, acute Q fever with positive antiphospholipid antibodies, and acute Q fever with underlying valvulopathy. Doxycycline is the antibiotic of choice in a dose of 100 mg orally b.i.d. [11]. The exact duration of treatment is unknown; some suggest the use of doxycycline for three days after apyrexia. If positive aCL, the authors previously mentioned recommend adding hydroxychloroquine for a minimum of three weeks and until normalization of the antiphospholipid antibodies. However, other authors suggest that antibiotic prophylaxis with a combination of doxycycline and hydroxychloroquine for a year was highly effective and prevented progression to endocarditis [12]. Hydroxychloroquine raises the pH of the phagolysosomes where *C. burnetii* replicates and allows more effective doxycycline activity [13].

The case was notified to public health authorities for further epidemiological investigation. Q fever is a mandatory notifiable disease in several countries.

## Conclusions

Our knowledge of the approach to Q fever remains incomplete, and adequate therapy is still under investigation, given the broad spectrum of the disease. aCL determination at the diagnosis of acute Q fever and during patient follow-up has recently been of greater use to prevent endocarditis, especially in men aged greater than 40 years. The addition of hydroxychloroquine to the treatment of acute Q fever in susceptible patients has proved to be effective in preventing progression to endocarditis.
